# Effects of Psychological Counselling Combined With Tolvaptan on Depression, Anxiety, and Quality of Life in Patients With Traumatic Brain Injury and Hyponatremia

**DOI:** 10.62641/aep.2234

**Published:** 2026-10-15

**Authors:** Yu Wang, Zhiwei Wang, Zhang Xiong, Hanyun Xu

**Affiliations:** ^1^Department of Neurosurgery, First Affiliated Hospital of Wannan Medical College, 241000 Wuhu, Anhui, China; ^2^Department of Neurosurgery, The Second Affiliated Hospital of Anhui Medical University, 230000 Hefei, Anhui, China

**Keywords:** depression, brain trauma, hyponatremia, tolvaptan, counseling

## Abstract

**Background:**

To explore the effects of psychological counselling model combined with tolvaptan on depression, anxiety, and quality of life in patients with traumatic brain injury (TBI) and hyponatremia.

**Methods:**

This retrospective study included 150 patients with TBI complicated by hyponatremia admitted to the First Affiliated Hospital of Wannan Medical College between May 2022 and May 2024. Based on the treatment methods provided in their medical records, the patients were divided into a combined treatment group (n = 75), receiving psychological counselling combined with tolvaptan, and a traditional treatment group (n = 75), receiving tolvaptan alone. Serum sodium levels, depression and anxiety status, quality of life, and self-efficacy were compared between the two groups before and after counselling.

**Results:**

Compared with the traditional treatment group, the combined treatment group showed better stability in serum sodium recovery, significantly lower depression and anxiety scores, and significantly higher scores in quality of life, self-efficacy, and social functioning than the traditional treatment group (all *p* < 0.05).

**Conclusions:**

Among patients with TBI and hyponatremia, psychological counselling combined with tolvaptan was associated with more stable serum sodium recovery, better emotional state and improved quality of life compared with tolvaptan alone. However, due to the retrospective nature of this study, these findings should be interpreted as correlational rather than causal. Prospective controlled studies are needed to further validate these findings.

## Introduction

Traumatic brain injury (TBI) is a major cause of disability and mortality among hospitalised neurosurgical patients. Its pathophysiology is complex and is often accompanied by neuroendocrine dysregulation and multisystem dysfunction. Water and electrolyte imbalances are particularly common during the acute and recovery phases of TBI, among which hyponatremia is one of the most representative clinical complications [1, 2, 3]. Hyponatremia is closely related to hypothalamic–pituitary axis dysfunction, dysregulated antidiuretic hormone secretion, and impaired volume regulation disorder. Persistent hyponatremia can not only aggravate cerebral oedema, but may also induce deeper consciousness disorders, epileptic seizures and delayed neurological function recovery, which will have an adverse effect on the overall prognosis of patients. With the improvement of neurocritical care and comprehensive treatment, the survival rate of patients with TBI has significantly improved, but the quality of functional recovery and long-term quality of life have gradually become the focus of clinical attention [4].

In clinical practice, patients with TBI and hyponatremia often experience varying degrees of depression and anxiety in addition to obvious physiological abnormalities [5]. The causes of this condition are not only related to the neurological damage caused by the disease itself, but also closely related to psychosocial factors such as the uncertainty of the disease course, functional limitations, long-term hospitalisation, and concerns about prognosis. Persistent negative emotional states can affect homeostasis through neuroendocrine and autonomic nervous system regulation pathways, thus potentially impacting electrolyte recovery and the overall rehabilitation process.

Tolvaptan, as a selective angiotensin V2 receptor antagonist, has a clear pharmacological basis for correcting dilutional hyponatremia, and its application in hyponatremia related to neurological diseases has gradually increased [6, 7, 8]. However, clinical practice suggests that correcting serum sodium levels alone is insufficient to improve patients’ functional recovery and quality of life. The role of factors such as emotional state, self-efficacy, and social function in the rehabilitation process of patients with TBI is receiving increasing attention. Psychological counselling, as a non-pharmacological management approach centred on emotional assessment, cognitive guidance, and supportive communication, has shown positive value in the management of various chronic and severe diseases [9, 10]. The importance of combination approaches over single-modality interventions has been increasingly recognised in clinical practice [11].

Existing research largely focuses on the pathogenesis and pharmacological treatment strategies of hyponatremia after TBI, particularly the efficacy of tolvaptan in correcting dilutional hyponatremia. In contrast, the impact of psychological and behavioural factors on electrolyte recovery and overall rehabilitation has received relatively little attention. Although mood disorders, decreased self-efficacy, and persistent psychological stress are common in patients with TBI, psychological management is often limited to empirical reassurance, lacking systematic evaluation and efficacy verification. Therefore, exploring the impact of psychological counselling on emotional state, quality of life, and rehabilitation-related outcomes under the background of standardised tolvaptan treatment is of significant practical importance.

Based on this, this study retrospectively analysed the clinical data of patients with TBI and hyponatremia between May 2022 and May 2024, comparing emotional state and quality of life between patients under different psychological counselling models in addition to tolvaptan treatment. The aim was to summarise valuable experiences from previous clinical practice and provide a more comprehensive reference for the integrated management of such patients. Unlike previous studies that focused solely on the pharmacological efficacy of tolvaptan or the psychological treatments alone, the present study evaluated the combined effect of these two approaches. It further explored whether adding systematic psychological counselling to standardised tolvaptan treatment was associated with benefits in emotional state, self-efficacy, and quality of life among patients with TBI and hyponatremia.

## Materials and Methods

### General Information

This retrospective study included 150 patients with TBI and hyponatremia who were hospitalised in the Department of Neurosurgery, First Affiliated Hospital of Wannan Medical College, between May 2022 and May 2024. Patients were divided into a combined treatment group (n = 75) and a traditional treatment group (n = 75) based on whether they received concurrent systematic psychological counselling as recorded in their medical records. This study was approved by the Medical Ethics Committee of First Affiliated Hospital of Wannan Medical College (Ethics Approval No.: 2024-186). Clinical data were retrospectively extracted from the electronic medical records system. Data was collected and analysed without interfering with the patient’s treatment. The study was conducted in accordance with the principles of the Declaration of Helsinki, and patient personal information was anonymised. All included patients or their legal representatives signed general informed consent forms during hospitalisation.

Consistent with clinical practice, systematic psychological counselling was only initiated after patients had achieved a Glasgow Coma Scale (GCS) score of ≥13 and were able to engage in basic verbal communication. [12, 13] The GCS scores at admission (reported in Table 1) were lower than this threshold in many patients, as expected, but all patients in the combined treatment group met these criteria before the counselling began. Baseline clinical data extracted from medical records included age, sex, GCS score at admission, time from injury to hospitalisation, and underlying diseases (hypertension, diabetes, and coronary heart disease).

### Inclusion and Exclusion Criteria

#### Inclusion Criteria

(a) Met the imaging and clinical diagnostic criteria for TBI [14]; (b) Had laboratory tests after admission confirmed hyponatremia (serum sodium <135 mmol/L), according to the diagnostic criteria of hyponatremia [1]; (c) Received tolvaptan treatment during hospitalisation; (d) Had complete medical records and traceable follow-up records; (e) Able to communicate and cooperate with basic psychological assessment.

#### Exclusion Criteria

(a) Patients with severe heart, liver, or kidney failure; (b) Patients with a previous diagnosis of mental disorder and long-term use of psychotropic medications. Anxiety or depressive symptoms secondary to TBI and hospitalisation were not an exclusion criterion, as they were the outcomes of interest in the present study; (c) Patients with malignant tumours or other end-stage diseases; (d) Patients with missing follow-up data or who were transferred to another hospital.

### Methods

#### Pharmacological Treatment

All enrolled patients received tolvaptan treatment according to their medical records. Tolvaptan tablets (H20213392, Nanjing Zhengda Tianqing Pharmaceutical Co., Ltd., Nanjing, Jiangsu, China) were administered orally at an initial dose of 7.5 mg once daily at a fixed time in the morning. Although the standard recommended initial dose for hyponatremia is 15 mg once daily, [15, 16] an initial dose of 7.5 mg is recommended for patients at risk of overly rapid correction of serum sodium or with severe hyponatremia [16]. In the present study, baseline serum sodium levels ranged from 125 to 129 mmol/L, and a cautious initial dose of 7.5 mg was therefore selected to minimise the risk of rapid sodium overcorrection, which is of particular concern in patients with TBI [17]. Based on changes in serum sodium and urine output, the dose was adjusted to 15 mg/day for some patients; all dose adjustments were documented in medical records. Serum sodium, electrolytes, and urine output were monitored daily during treatment, and fluid restriction measures were provided as needed.

#### Traditional Treatment Group

(1) Patient condition monitoring: According to the routine treatment protocol of neurosurgery, the patient’s consciousness, pupil changes and limb movement were assessed and recorded at regular intervals; vital signs, including body temperature, pulse, respiration rate and blood pressure, were monitored daily.

(2) Electrolyte and fluid intake management: Daily blood sodium and electrolyte related tests were performed as clinically indicated. Urine output, water intake and intravenous fluid infusion were recorded, and water restriction or fluid replacement measures was implemented according to the physician’s orders to maintain appropriate fluid balance.

(3) Medication guidance: Tolvaptan and other prescribed medications were administered according to the physician’s orders, and patients were monitored to take their medication on time. After taking the medication, patients were observed for obvious thirst, changes in urine output, and any abnormalities were recorded and reported in a timely manner.

(4) Basic health education: Routine verbal education were given to patients and their families on a daily basis, including the name of the disease, precautions for hospitalisation, requirements for cooperation with examinations and safety precautions.

#### Combined Treatment Group

In addition to conventional treatment, patients in the combined treatment group received structured psychological counselling focusing on their emotional status and psychological responses. The psychological counselling was delivered by trained clinical psychologists (master’s degree with ≥3 years of experience in neuropsychological rehabilitation). Each session lasted 30–45 minutes and was delivered once daily during the first week, followed by once every other day thereafter. Treatment fidelity was ensured through weekly supervision meetings and review of session recordings for 20% of randomly selected cases. In accordance with routine clinical practice [12, 13], psychological counselling was initiated only after patients had achieved a GCS score of ≥13 (usually 3–5 days after admission).

The treatment protocol comprised the following six components:

(1) Supportive psychological guidance: During the hospitalisation process, continuous doctor-patient communication was used to provide emotional support to patients, guiding them to express their true feelings about the disease, treatment process and prognosis. This guidance emphasised listening and empathy, avoiding value judgments or over-interpretations of patients’ emotions, so that they could have a subjective experience of being understood and accepted, thereby relieving tension and anxiety.

(2) Cognitive clarification and information provision: In response to the fear and misunderstanding related to hyponatremia, changes in urine volume or fluctuations in test results, relevant medical information was explained in a targeted manner in combination with the actual diagnosis and treatment progress to help patients form an objective understanding of their current condition. Patients’ understanding of the disease status and treatment goals was reinforced throughout the intervention to reduce the psychological stress caused by information uncertainty.

(3) Emotion recognition and emotion focus: Patients were monitored for emotion changes and assisted in identifying the specific sources of anxiety and depression, such as pain, sleep disorders or concerns about the prognosis. By guiding patients to concretize vague emotional experiences and decompose and process current manageable real-world problems, the emotional distress and persistent psychological tension can be reduced.

(4) Self-efficacy enhancement: Patients were guided to actively participate in their own treatment process, so that their sense of control over disease management and rehabilitation can be strengthened. Positive feedback was given when patients completed medication, cooperated with examinations, or when their conditions were relatively stable, to help them establish a subjective cognition of “achievable and improvable”. At the same time, patients were guided to use short relaxation methods, such as rhythmic breathing adjustment, to improve tension and enhance self-regulation ability.

(5) Family support guidance: Brief communication guidance was given to their main family members, emphasising positive and stable communication methods, and avoiding reinforcing the patient’s excessive focus on the uncertainty of the disease.

(6) Environmental and rhythmic counselling: Based on the patient’s condition and emotional characteristics, the hospital environment and daily routine was adjusted to minimise unnecessary sensory stimulation and maintain a relatively stable diurnal rhythm. Patients were encouraged to participate in daily activities within their capabilities when their conditions allow, to enhance their sense of participation in life and restore confidence. All psychological counselling sessions were documented using a standardised checklist to ensure consistency across patients.

#### Treatment Duration

All patients received treatment during their entire hospitalisation period. The mean length of hospital stay was 14.2 ± 3.5 days for the combined treatment group and 13.8 ± 4.1 days for the traditional treatment group (*p* = 0.512), ensuring comparable treatment duration between groups.

### Observation Indicators

All baseline assessments were performed within 24 hours of admission, and pre-discharge assessments were conducted within 48 hours before discharge.

#### Analysis of Serum Sodium Levels Before and After Treatment

Serum sodium levels were measured from fasting venous blood samples collected at pre-counseling and post-counseling using standard laboratory methods in the hospital’s clinical laboratory. Venous blood samples were drawn in the morning after an overnight fast.

#### Assessment for Depression and Anxiety

The 17-item Hamilton Depression Scale (HAMD-17) [18] was used to assess patients’ depressive state. It consists of 17 items, with most items scored from 0 to 4 points and a few from 0 to 2 points. A higher total score indicates a more severe degree of depression. The Hamilton Anxiety Scale (HAMA) [19] was used to assess patients’ anxiety state. It consists of 14 items, each scored from 0 to 4 points, with a total score of 0–56 points. A higher score indicates a more pronounced degree of anxiety.

#### Assessment for Psychological Stress Level

The Perceived Stress Scale (PSS-10) [20] was used to assess patients’ subjective psychological stress level. This scale consists of 10 items, each scored from 0 to 4 points, with a total score of 0–40 points. Higher scores indicate higher perceived stress levels.

#### Self-Efficacy Assessment

The General Self-Efficacy Scale (GSES) [21] was used to assess patients’ self-management and coping abilities. The scale consists of 10 items, each scored from 1 to 4 points, yielding a total score of 10–40 points. Higher scores indicate higher levels of self-efficacy.

#### Quality of Life Assessment

The Generic Quality of Life Inventory-74 (GQOL-74) [22] scale was used to assess patients’ quality of life. The scale includes four dimensions: physical function, psychological function, social function, and material life status. Each dimension has a score range of 0–100 points. Higher scores indicate better quality of life.

#### Internal Consistency Reliability of Scales

Based on the data of the 150 participants in the present study, the Cronbach’s α values for the scales were as follows: HAMD-17 (α = 0.82), HAMA (α = 0.81), PSS-10 (α = 0.83), GSES (α = 0.85), and GQOL-74 (α = 0.87 for the total score). These values indicate acceptable internal consistency for all scales in this study population.

### Statistical Methods

No a priori sample size calculation was performed, as all eligible patients were consecutively included during the study period.

Data analysis was performed using SPSS 26.0 statistical software (IBM Corp., Armonk, NY, USA). Normality of continuous variables was tested using the Shapiro–Wilk test, and homogeneity of variances was tested using Levene’s test. Data that met these assumptions were analysed using parametric tests. If violated, Mann–Whitney U test was applied. Normally distributed continuous data are presented as mean ± standard deviation ($\bar{x}$ ± s), and comparisons between two groups were performed using independent-sample *t*-tests. Categorical data are presented as counts (percentages) and were compared using the χ^2^ test or Fisher’s exact test, as appropriate.

For repeated measures data (serum sodium levels, HAMD, HAMA, PSS-10, GSES, and GQOL-74 scores), within-group comparisons (pre-counselling vs. post-counselling) were performed using paired *t*-tests. Between-group comparisons of post-counselling outcomes were performed using analysis of covariance (ANCOVA) with the pre-counselling GCS score included as a covariate to adjust for baseline neurological status. A two-tailed *p*-value < 0.05 was considered statistically significant.

## Results

### Comparison of Baseline Data Between the Two groups

There were no significant differences between the two groups in terms of GCS scores, age, sex, time from injury to admission, and underlying diseases (hypertension, diabetes, coronary heart disease) (all *p* > 0.05). Before the first psychological counselling session, all patients had GCS scores ranging from 13 to 15, meeting the predefined criterion of GCS ≥13 for psychological counselling (Table 1).

**Table 1.** **Baseline data of the two groups**.

Group	n	Sex		Age	GCS score	Time from injury	Underlying diseases		
		Male	Female	(years)	(points)	to hospitalisation	Hypertension	Diabetes	Coronary heart disease
Combined	75	42	33	46.32 ± 11.21	14.25 ± 0.68	4.62 ± 1.87	19	13	9
treatment group									
Traditional	75	40	35	45.79 ± 10.86	14.29 ± 0.56	4.78 ± 1.92	21	12	10
treatment group									
*t*/χ^2^		0.108		0.294	–0.392	0.517	0.136		
*p*		>0.05		>0.05	>0.05	>0.05	>0.05		

Note: Pre-counselling GCS scores were assessed before the first counselling session. GCS, Glasgow Coma Scale.

### Comparison of Serum Sodium Levels Before and After Treatment in the Two Groups

There was no significant difference in pre-counselling serum sodium levels between the two groups (*p* > 0.05). The post-counselling serum sodium level in the combined treatment group was significantly higher than that in the traditional treatment group (*p* < 0.05). After adjusting for pre-counselling GCS score, ANCOVA confirmed that the combined treatment group had significantly higher post-counselling serum sodium levels than the traditional treatment group (adjusted mean: 137.26 vs. 134.81 mmol/L; mean difference = 2.45, 95% confidence interval (CI): 1.47–3.44; F(1,147) = 24.16, *p* < 0.001, partial η^2^ = 0.141). Intra-group comparisons showed that post-counselling serum sodium level was significantly higher than pre-counselling level in the combined treatment group (*p* < 0.05), and serum sodium level before discharge was also higher than the level at admission in the traditional treatment group (*p* < 0.05), with a lower increase than the combined treatment group (Table 2).

**Table 2.** **Comparison of serum sodium levels before and after treatment in the two groups (mmol/L, $\bar{x}$ ± s)**.

Group	n	Pre-counselling	Post-counselling	ANCOVA adjusted mean (95% CI)
Combined treatment group	75	129.14 ± 3.82	136.15 ± 2.84*	137.26 (136.57, 137.96)
Traditional treatment group	75	129.42 ± 3.76	134.63 ± 3.02*	134.81 (134.11, 135.51)
Mean difference (95% CI)				2.45 (1.47, 3.44)
*t*/F		0.452	3.176	24.16
*p*		>0.05	<0.05	<0.001

Note: Compared with the pre-counselling data in this group, * *p* < 0.05. CI, confidence interval; ANCOVA, analysis of covariance.

### Comparison of Depression and Anxiety States Between the Two Groups

After the treatment, the depression and anxiety scores in the combined treatment group were significantly lower than those in the traditional treatment group (all *p* < 0.05). After adjusting for pre-counselling GCS, ANCOVA showed that the combined treatment group had significantly lower post-counselling HAMD and HAMA scores than the traditional treatment group. For HAMD, the adjusted mean (standard error [SE]) was 9.16 vs. 11.94, mean difference = −2.78 (95% CI: −3.68 to −1.88, F(1,147) = 37.41, *p* < 0.001, partial η^2^ = 0.203). For HAMA, the adjusted mean (SE) was 9.60 vs. 11.19, mean difference = −1.59 (95% CI: −2.27 to −0.91, F(1,147) = 21.26, *p* < 0.001, partial η^2^ = 0.126). Intra-group comparisons showed that the post-counselling HAMD and HAMA scores in the combined treatment group decreased significantly compared with pre-counselling scores (*p* < 0.05); the post-counselling HAMD and HAMA scores of the traditional treatment group also decreased compared with pre-counselling scores (*p* < 0.05), with a smaller decrease than the combined treatment group (Table 3).

**Table 3.** **Comparison of anxiety and depression scores between the two groups (points)**.

Group	n	HAMD			HAMA		
		Pre-counselling	Post-counselling	ANCOVA adjusted mean (95% CI)	Pre-counselling	Post-counselling	ANCOVA adjusted
Combined	75	18.42 ± 4.31	9.26 ± 3.18*	9.16 (8.52, 9.79)	19.38 ± 4.56	9.11 ± 3.04*	9.60 (9.12, 10.08)
treatment group							
Traditional	75	18.17 ± 4.09	12.84 ± 3.67*	11.94 (11.30, 12.57)	19.22 ± 4.48	11.29 ± 3.11*	11.19 (10.71, 11.67)
treatment group							
Mean difference				–2.78 (–3.68, –1.88)			–1.59 (–2.27, –0.91)
(95% CI)							
*t*/F		0.364	6.385	37.41	0.422	4.341	21.26
*p*		>0.05	<0.05	<0.001	>0.05	<0.05	<0.001

Note: Compared with the pre-counselling data in this group, * *p* < 0.05. HAMD, Hamilton Depression Scale; HAMA, Hamilton Anxiety Scale; CI, confidence interval; ANCOVA, analysis of covariance.

### Comparison of PSS-10 Scores Between the Two Groups

Both groups showed significant decreases in PSS-10 scores between post-counselling and pre-counselling (both *p* < 0.05), with a more significant decrease in the combined treatment group. The post-counselling PSS-10 score in the combined treatment group was significantly lower than that in the traditional treatment group (*p* < 0.05). After adjusting for pre-counselling GCS score, ANCOVA confirmed that the combined treatment group had significantly lower post-counselling PSS-10 scores than the traditional treatment group (adjusted mean: 13.07 vs. 16.48 points; mean difference = −3.41, 95% CI: −4.08 to −2.75; F(1,147) = 52.30, *p* < 0.001, partial η^2^ = 0.265) (Table 4).

**Table 4.** **Comparison of PSS-10 scores between the two groups (points)**.

Group	n	Pre-counselling	Post-counselling	ANCOVA adjusted mean (95% CI)
Combined treatment group	75	22.81 ± 4.96	13.42 ± 3.71*	13.07 (12.60, 13.54)
Traditional treatment group	75	22.39 ± 5.04	16.87 ± 4.09*	16.48 (16.01, 16.95)
Mean difference (95% CI)				–3.41 (–4.08, –2.75)
*t*/F		0.514	5.411	52.30
*p*		>0.05	<0.05	<0.001

Note: Compared with the pre-counselling data in this group, * *p* < 0.05. CI, confidence interval; ANCOVA, analysis of covariance; PSS-10, Perceived Stress Scale.

### Comparison of GSES Scores Between the Two Groups

Intra-group comparisons showed that the post-counselling GSES scores of both groups were higher than the pre-counselling scores (*p* < 0.05), with the combined treatment group showing a significantly greater increase than the traditional treatment group. The GSES scores of the combined treatment group were significantly higher than those of the traditional treatment group at discharge (*p* < 0.05). After adjusting for pre-counselling GCS score, ANCOVA confirmed that the combined treatment group had significantly higher post-counselling GSES scores than the traditional treatment group (adjusted mean: 28.56 vs. 24.64 points; mean difference = 3.92, 95% CI: 2.95 to 4.89; F(1,147) = 64.00, *p* < 0.001, partial η^2^ = 0.303) (Table 5).

**Table 5.** **Comparison of GSES scores between the two groups (points)**.

Group	n	Pre-counselling	Post-counselling	ANCOVA adjusted mean (95% CI)
Combined treatment group	75	19.34 ± 4.17	28.62 ± 3.94*	28.56 (27.88, 29.24)
Traditional treatment group	75	19.71 ± 4.08	24.81 ± 4.12*	24.64 (23.96, 25.33)
Mean difference (95% CI)				3.92 (2.95, 4.89)
*t*/F		0.549	5.788	64.00
*p*		>0.05	<0.05	<0.001

Note: Compared with the pre-counselling data in this group, * *p* < 0.05. CI, confidence interval; ANCOVA, analysis of covariance; GSES, General Self-Efficacy Scale.

### Comparison of Quality of Life Between the Two Groups

Intra-group comparisons showed that the post-counselling GQOL-74 scores of both groups were higher than the pre-counselling scores (*p* < 0.05). The post-counselling GQOL-74 scores in the combined treatment group were significantly higher than those in the traditional treatment group (*p* < 0.05). After adjusting for pre-counselling GCS score, ANCOVA revealed that the combined treatment group had significantly higher post-counselling scores in all four dimensions of the GQOL-74. For material life, the adjusted mean was 73.15 vs. 61.44 points (mean difference = 11.68, 95% CI: 10.61 to 12.76; F(1,147) = 68.25, *p* < 0.001, partial η^2^ = 0.317). For social function, adjusted mean = 72.07 vs. 65.46 points (mean difference = 6.61, 95% CI: 5.47 to 7.76; F(1,147) = 48.62, *p* < 0.001, partial η^2^ = 0.249). For somatic functions, adjusted mean = 71.69 vs. 63.98 points (mean difference = 7.70, 95% CI: 6.44 to 8.97; F(1,147) = 53.71, *p* < 0.001, partial η^2^ = 0.270). For psychological function, adjusted mean = 72.12 vs. 62.53 points (mean difference = 9.59, 95% CI: 8.41 to 10.77; F(1,147) = 61.34, *p* < 0.001, partial η^2^ = 0.295) (Table 6).

**Table 6.** **Comparison of GQOL-74 scores between the two groups (points)**.

Group	n	Material life			Social function			Somatic functions			Psychological function		
name		Pre-	Post-	ANCOVA	Pre-	Post-	ANCOVA	Pre-	Post-	ANCOVA	Pre-	Post-	ANCOVA
		counselling	counselling	adjusted mean	counselling	counselling	adjusted mean	counselling	counselling	adjusted mean	counselling	counselling	adjusted mean
				(95% CI)			(95% CI)			(95% CI)			(95% CI)
Combined	75	60.84 ± 5.11	73.44 ± 5.27*	73.15	61.27 ± 4.96	72.29 ± 5.14*	72.07	62.13 ± 5.38	71.72 ± 6.03*	71.69	60.92 ± 5.24	72.43 ± 5.04*	72.12
treatment				(72.38, 73.89)			(71.26, 72.88)			(70.79, 72.58)			(71.29, 72.96)
group													
Traditional	75	61.02 ± 5.07	61.18 ± 4.42*	61.44	60.88 ± 5.02	65.88 ± 4.03*	65.46	61.76 ± 5.44	64.26 ± 5.24*	63.98	61.13 ± 5.31	63.11 ± 6.97*	62.53
treatment				(60.69, 62.21)			(64.65, 66.27)			(63.09, 64.88)			(61.70, 63.37)
group													
*t*/F		0.217	11.237	68.25	0.479	6.207	48.62	0.419	5.906	53.71	0.244	6.853	61.34
*p*		>0.05	<0.05	<0.001	>0.05	<0.05	<0.001	>0.05	<0.05	<0.001	>0.05	<0.05	<0.001

Note: Compared with the pre-counselling data in this group, * *p* < 0.05. GQOL-74, the Generic Quality of Life Inventory-74; CI, confidence interval; ANCOVA, analysis of covariance.

## Discussion

In addition to complex neurological damages, patients with TBI and hyponatremia usually experience significant electrolyte disturbances as well as psychological and behavioural changes [23]. This study found that patients receiving systematic psychological counselling based on tolvaptan treatment showed more favourable trends in multiple indicators such as serum sodium stability, emotional state, self-efficacy and quality of life. These findings suggest an association between the psychological counselling and improvements in psychological state and rehabilitation-related indicators.

Since hyponatremia is a common electrolyte abnormality after TBI, its pathogenesis involves factors such as abnormal secretion of antidiuretic hormone, hypothalamic–pituitary regulatory dysfunction and fluid regulation imbalance [24]. In this study, the serum sodium level in the combined treatment group was higher than that in the traditional treatment group before discharge (*p* < 0.05). A possible explanation for this observation is that emotional tension and anxiety may influence humoral regulation through sympathetic excitation and endocrine stress response [25]. However, an alternative interpretation is that patients who received systematic psychological counselling also had better treatment adherence (including fluid management), which could directly contribute to serum sodium improvement [26].

In addition, the HAMD-17 and HAMA scores in the combined treatment group were significantly lower than those in the traditional treatment group before discharge (*p* < 0.05). Since the sources of negative emotions in patients with TBI are multifaceted, including uncertainty about the recovery of neurological function, concerns about long-term prognosis, and psychological stress from the hospital environment, the combined treatment group received more continuous emotional assessment, emotional expression support, and non-verbal psychological relief measures during hospitalisation. These measures not only provide emotional comfort but also reduce patients’ sustained psychological arousal level by stabilising environmental stimuli, reducing unnecessary sensory load, and improving sleep rhythm, thereby helping to alleviate depression and anxiety.

The present study also showed that the PSS-10 score in the combined treatment group was lower than that in the traditional treatment group (*p* < 0.05), because the patients in the combined treatment group received clearer information interpretation during the stages of disease changes, examination result updates and treatment adjustments. This continuity of information helps to reduce the patients’ vigilance to unpredictable events, thereby reducing persistent psychological stress response. Meanwhile, self-efficacy is a comprehensive evaluation of a patient’s ability to cope with illness. The GSES results showed that the self-efficacy of the combined treatment group was significantly higher than that of the traditional treatment group (*p* < 0.05). This finding may reflect that patients in the combined treatment group had greater opportunities for participation and positive feedback, which could contribute to an enhanced sense of control over their recovery process, such as eating independently, cooperating with examinations and bedside activities. The accumulation of this sense of participation enabled the patients to gradually form a subjective experience that they could complete and control, thereby enhancing their confidence in recovery process. This is reflected in the GSES score as an improvement in self-efficacy level.

Furthermore, the GQOL-74 score in the combined treatment group was higher than that in the traditional treatment group (*p* < 0.05). The combined treatment group formed a relatively continuous emotional stability process during hospitalisation, which reduced the patients’ uncertainty and threat perception of the disease, thereby obtaining a higher score in the psychological function dimension. Nevertheless, the observational design precludes definitive causal conclusions. In addition, self-efficacy, as an individual’s subjective judgment of their own coping ability, directly affects their willingness to participate in society and their role adaptation ability. Patients in the combined treatment group had more opportunities to participate and receive positive feedback, and gradually established a sense of control over their own recovery process. Moreover, through environmental adjustment, rhythm management and relaxation training measures, the above comprehensive measures help to improve the patients’ quality of life.

### Limitations

Several limitations of this study should be acknowledged. First, the retrospective, non-randomised design introduces inherent selection bias. Patients who received psychological counselling may have differed from those who did not in unmeasured variables such as motivation, family support, or baseline neurological status, despite the GCS adjustment. Second, the single-centre design limits generalisability. Third, the lack of objective biomarkers (e.g., antidiuretic hormone [ADH] levels, detailed fluid balance records) and medication adherence monitoring prevents a definitive mechanistic explanation for the observed serum sodium improvement. Fourth, the short-term observation period (hospitalisation to discharge) cannot capture long-term psychological or functional outcomes. Fifth, the psychological counselling treatment, although structured, was not manually controlled, and inter-provider variability may exist. Therefore, the findings should be interpreted as exploratory and hypothesis-generating rather than confirmatory. Prospective, multicenter, randomised controlled trials are needed to validate the observed associations. Additionally, the specific subtypes of hyponatremia (e.g., syndrome of inappropriate antidiuretic hormone secretion [SIADH] vs. cerebral salt‑wasting syndrome [CSWS]) were not systematically recorded.

## Conclusions

This retrospective study found that TBI patients with hyponatremia who received systematic psychological counselling in addition to tolvaptan treatment showed better serum sodium stability, lower depression and anxiety scores, and higher self-efficacy and quality of life scores compared with those receiving tolvaptan treatment alone. These findings suggest a potential association between the combined approach and improved psychological and physiological outcomes. However, given the retrospective design, causal conclusions cannot be drawn. The combined strategy may warrant further investigation in prospective, randomised controlled trials to validate its clinical value.

## Availability of Data and Materials

The datasets used and/or analyzed during the current study were available from the corresponding author on reasonable request.

## References

[1] Spasovski G, Vanholder R, Allolio B, Annane D, Ball S, Bichet D (2014). Clinical practice guideline on diagnosis and treatment of hyponatraemia. *European journal of endocrinology*.

[2] Yang Y, Niu L (2022). Effect of Early Rehabilitation Nursing on Motor Function and Living Ability of Patients with Traumatic Brain Injury Based on Orem’s Self-Care Theory. *Computational intelligence and neuroscience*.

[3] Tomkins M, Green D, O’Reilly MW, Sherlock M (2025). Fluid and electrolyte disorders following traumatic brain injury. Best practice & research. *Clinical endocrinology & metabolism*.

[4] Hannon MJ, Thompson CJ (2014). Neurosurgical Hyponatremia. *Journal of clinical medicine*.

[5] Léveillé E, Aljassar M, Beland B, Saeedi RJ, Marcoux J (2022). Determinants of hyponatremia following a traumatic brain injury. *Neurological sciences: official journal of the Italian Neurological Society and of the Italian Society of Clinical Neurophysiology*.

[6] Rauwenhoff JCC, Bol Y, Peeters F, Smits P, Duits A, Wijenberg M (2024). Acceptance and commitment therapy for people with depressive and anxiety symptoms following acquired brain injury: Results of the BrainACT randomized controlled trial. *Journal of psychosomatic research*.

[7] Ma CY, Hsiao YW, Tzeng HY, Hueng DY, Chiang HH (2024). Relations between concussion symptoms and depression among patients with mild traumatic brain injury: A moderated mediation model. *Journal of nursing scholarship: an official publication of Sigma Theta Tau International Honor Society of Nursing*.

[8] Petria I, Indirli R, Mantovani B, Lanzi V, Mantovani G, Ferrante E (2025). Recurrent hyponatremia due to syndrome of inappropriate antidiuresis after traumatic brain injury: two case reports. *Frontiers in endocrinology*.

[9] Sander AM, Clark AN, Arciniegas DB, Tran K, Leon-Novelo L, Ngan E (2021). A randomized controlled trial of acceptance and commitment therapy for psychological distress among persons with traumatic brain injury. *Neuropsychological rehabilitation*.

[10] Sathananthan N, Morris EMJ, Gillanders D, Knox L, Dimech-Betancourt B, Wright BJ (2022). Does Integrating Cognitive and Psychological Interventions Enhance Wellbeing After Acquired Brain Injury? Study Protocol for a Phase II Randomized Controlled Trial of the VaLiANT (Valued Living After Neurological Trauma) Group Program. *Frontiers in rehabilitation sciences*.

[11] Sonkin D, Thomas A, Teicher BA (2024). Cancer treatments: Past, present, and future. *Cancer genetics*.

[12] Teasdale G, Jennett B (1974). Assessment of coma and impaired consciousness. A practical scale. *Lancet*.

[13] Mikolić A, Shi S, Panenka W, Brubacher JR, Scheuermeyer FX, Nelson LD (2025). Outcomes after Traumatic Brain Injury with and Without Computed Tomography. *Journal of neurotrauma*.

[14] Menon DK, Silverberg ND, Ferguson AR, Bayuk TJ, Bhattacharyay S, Brody DL (2025). Clinical Assessment on Days 1-14 for the Characterization of Traumatic Brain Injury: Recommendations from the 2024 NINDS Traumatic Brain Injury Classification and Nomenclature Initiative Clinical/Symptoms Working Group. *Journal of neurotrauma*.

[15] Otsuka Pharmaceutical Co., Ltd (2009). Samsca (tolvaptan) prescribing information.

[16] Dr. Reddy’s Laboratories (UK) Ltd (2025). Tolvaptan 7.5 mg tablets: summary of product characteristics.

[17] Onuigbo MAC, Agbasi N (2016). Severe symptomatic acute hyponatremia in traumatic brain injury responded very rapidly to a single 15 mg dose of oral tolvaptan; a Mayo Clinic Health System hospital experience – need for caution with tolvaptan in younger patients with preserved renal function. *Journal of renal injury prevention*.

[18] Hamilton M (1960). A rating scale for depression. *Journal of neurology, neurosurgery, and psychiatry*.

[19] Hamilton M (1959). The assessment of anxiety states by rating. *The British journal of medical psychology*.

[20] Cohen S, Kamarck T, Mermelstein R (1983). A global measure of perceived stress. *Journal of health and social behavior*.

[21] Schwarzer R, Jerusalem M, Weinman J, Wright S, Johnston M (1995). Generalized Self-Efficacy scale. *Measures in Health Psychology: A User’s Portfolio: Causal and Control Beliefs*.

[22] Qian X, Pan K, Liang C, Chen H, Hui J (2024). Osteoporotic thoracolumbar spine fractures in the elderly: alterations in GNRI and BMP-2 in delayed union and associated factors. *American journal of translational research*.

[23] Oyesanya TO, Harris G, Cary MP Jr, Byom L, Yang Q, Bettger JP (2021). Age- and sex-specific predictors of inpatient rehabilitation facility discharge destination for adult patients with traumatic brain injury. *Brain injury*.

[24] Smith JA, Pati D, Wang L, de Kloet AD, Frazier CJ, Krause EG (2015). Hydration and beyond: neuropeptides as mediators of hydromineral balance, anxiety and stress-responsiveness. *Frontiers in systems neuroscience*.

[25] Light KC, Koepke JP, Obrist PA, Willis PW 4th (1983). Psychological stress induces sodium and fluid retention in men at high risk for hypertension. *Science (New York, N.Y.)*.

[26] Zolnierek KB, Dimatteo MR (2009). Physician communication and patient adherence to treatment: a meta-analysis. *Medical care*.

